# Clinical Report

**Published:** 1860-09

**Authors:** N. S. Davis


### CLINICAL REPORT.

Medical Wards of Mercy Hospital. Service of Dr. N. S. Davis.

Reported by C. DUMREICHER.

The time this morning for clinical instruction, was occupied with two cases, one of Chronic Dysentery, and one of Continued Fever. Mr. ———, aged ——, was admitted to the Hospital. He had been suffering from dysentery for more than a year. He had been for some time under treatment in one of the Hospitals of New Orleans, and subsequently in a

Hospital in this city with only temporary benefit. From the character of the discharge, it was probable, that the inflammation extended from the upper part of the Colon into the lower part of the Ilium. The case gave rise to a more close and careful inquiry into the different pathological conditions, constituting chronic dysentery and diarrhœa. The first and most frequent condition was stated to be simple inflammation of the mucous membrane of the intestines, causing in its more chronic form, a thickened and indurated condition of the mucous membrane, and resulting in ulceration, which attacks any part of the intestinal canal indiscriminately.

The attention of the class was called to the difference in the pathological appearances of the intestinal canal in dysentery, and in typhoid fever. It was remarked, that while in typhoid fever the ulceration is limited to the glandular structures, and involving almost always the mesenteric glands, in dysentery this ulceration extends indiscriminately to all the structures of the intestinal surface, and attacks very seldom the mesenteric glands.

As a second condition giving rise to chronic dysentery, was enumerated local ulceration of the intestinal canal, not unfrequently the sequal of typhus and typhoid fevers. In these cases, it will almost always be found, that enlargement and softening of the mesenteric glands has taken place also.

Tubercular deposit in the mucous membrane of the intestinal canal, was given as the third and most fatal condition, giving rise to dysentery. If the disease resists all rational treatment, and particularly if connected with tubercular deposits in the lungs, we may confidently predict the existence of tubercular deposits, situated in the mucous follicles of the intestines. In many of these cases the patient will not complain of any cough, or only very little ; but this ought not to mislead the practitioner, as a perverted sensibility accounts sufficiently for it, while by the aid of the physical signs, auscultation and percussion, he is enabled to determine with correctness and certainty the presence of a disease of that kind. The case showed very plainly how necessary it was to understand the true pathology of these cases, to give a correct prognosis.

The history of the present case indicated that it belonged to the third of the above divisions. A careful examination proved it to be so. The space immediately beneath the clavicle, appeared to be sunken ; the inspiratory murmur was short, while the expiratory was rather prolonged ; the increase of the vibration of voice was strongly marked, indicating an atrophied condition of the upper part of the lung, with increased density. The condition of the blood has become considerably changed ; the corpuscles are diminished in number, while the watery portion is relatively increased.

The indications for treatment, are according to the pathology given above, unmistakable. First endeavor to keep the bowels as quiet as possible, by diminishing the peristalic action of the intestinal canal, and allaying the morbid sensitiveness of the involved parts. Second, to promote a more ready cicatrization where ulceration has taken place. Third, to improve the quality of the blood. The nitr. silv. was recommended in combination with opium, to accomplish the two first objects. The sulph. of iron and copper were mentioned for the same purpose. Acting differently, but yet designed to accomplish the same objects, were mentioned the oil of Turpentine and the gum Benzoin, given with opium prepared in the form of emulsions. The sulph. of Iron and subnitr. of Bismuth were alluded to as sometimes valuable for increasing digestion by invigorating the stomach.

During the two proceeding days, the patient has been taking a pill every two hours, containing Nit. Argent ⅛ gr., and Pulv. Opii. 1 gr. At present this pill was directed to be continued every four hours, and alternated with a teaspoonful of the following emulsion, viz :

| | | |
|---|---|---|
| ℞ | Pulv. G. Benzion, | ʒ ii. |
| | Tinc. Opii. | ʒ ii. |
| | Pulv. G. Arabac, White Sugar, | aa. ʒ iii. |
| | Mint. Water, | ℥ ii. |

Rub together for an emulsion.

For nourishment, the patient was advised to live exclusively on Milk with Lime Water, or Milk Porridge. This course of treatment was continued for two days with considerable relief, but at the end of that time the discharges became more frequent; and the following powder was substituted for the pill of Nit. Argent, and Opium, viz:

| | | |
|---|---|---|
| ℞. | Oxide of Zinc, | 3 qrs. |
| | Tannate Quinine, | 2 qrs. |
| | Pulv. Opii. | 1 gr. |

Mix one powder.

The same nourishment was continued. After continuing these powders alternated with the emulsion three days, the discharges had become less frequent and painful, but the patient was much debilitated. The emulsion was then omitted, and the Liquor Ferri. Nitratis 15 gtts. given every four hours in its place, the powders being continued, and same nourishment as before.

This treatment has been continued until the present time, (three weeks since the patient was admitted,) and his discharges are reduced to about three in 24 hours, are more natural, and the patient has gained decidedly in flesh and strength.

---

*Typhoid Fever.—Pulmonary Engorgement.*—The second case to which our attention was called during the clinic hour, was that of a German aged about 18 years. He had been admitted into the Hospital ten days previously. At the time of admission he presented all the ordinary phenomena of an idiopathic fever. His skin was hot and dry; his face flushed with a diffused redness; his expression dull and mind gloomy; his tongue covered with a dirty white coat; sore throat; pulse 110 per minute, but not full; bowels inactive; with much dull aching pain in the head, back and limbs. All these symptoms were much diminished during each morning, constituting a well marked remission. But there was neither a well marked chill nor a sweating stage. The attention of the clinical class was directed to the case at that period of its progress, as one presenting the interesting questions of diagnosis between paroxysmal or *exascerbating* cases of continued fever, and those of true *remittent* or malarious fever. It was remarked that many cases of continued fever, during the early part of their progress, present such distinct daily exascerbations and remissions, that they sometimes lead even the experienced practitioner into doubt, as to whether they are actual cases of continued fever or genuine remittents. If such cases are scanned closely, however, it will be found that the exascerbating continued fever patient always presents a dullness of expression, a dingy suffused redness of the face, red and dry lips, obtuseness of the mental faculties, and a quick soft pulse, which is widely different from the more anxious and active expression of countenance, the bright flush on the cheeks, the acuteness of the special senses, the more full and active pulse, the more sallow hue of the skin, and more frequent billious vomiting, that generally accompanies the paroxysm of a Remittent. Although Prof. Davis remarked at the first interview that the present case would doubtless prove to be one of Typhoid Fever, yet as it was plainly exascerbating and many practitioners thought Quinine valuable, to say the least, harmless, he would prescribe the following course of treatment.

℞ Spts. Nit. Dulc., ℥ i.

Qinct. Opii. et Camph., ℥ i.

Tinct. Verat. Viride, ʒ i.

Mix, and give a teaspoonful every three hours during the afternoon and evening while the febrile exascerbation continues.

℞ Sulph Quinine, 12 grs.
Pulv. Opii., 3 grs.
Blue Mass, 6 grs.

Mix, divide into three powders, and give one at 5, 8 and 11 o'clock, A. M., constituting the period of remission. This course was followed for three days in succession, when the active febrile exascerbations ceased, the pulse became slower, the headache ceased, and all the phenomena of fever were diminished, but at the same time the bowels began to move too frequently, the discharges being thin and brown. Both the previous prescriptions were now omitted, and the following emulsion ordered in doses of a teaspoonful, with two grains of Sulphate of Quinine added to each dose when taken.

℞ Ol. Terebinth, ʒ ii.

Tinct. Opii., ʒ ii.

Pulv. G. Arabac, } aa. ʒ iii.

White Sugar, }

Rub together and add

Mint Water, ℥ ii. Mix.

This checked the intestinal discharges, and for two days the symptoms indicated a speedy convalescense. At the end of that time, however, the tongue became more dry and brown; the mind of the patient constantly wandering; the pulse quicker; the skin more dry; the bowels slightly tympanitic; and still two or three liquid brown stools in the 24 hours. There was also noticed at that time an occasional dry bronchial cough with dry bronchial rales on both sides of the chest. To counteract these symptoms the emulsion was continued, and 15 drops of chloroform given between each of the doses. The patient was also allowed to take pretty freely of a solution of chlorate of Potassa. Notwithstanding these remedies, the lungs became every day more engorged, and the breathing in consequence more noisy and difficult; the bowels continued to move three or four times a day; the hearing became dull; the delirium constant; some sub-sultus; the pulse soft and frequent; and the impulse of the heart feeble. At the suggestion of another the patient was allowed to take brandy punch freely, in addition to the other remedies, but instead of ameliorating the condition of the patient, thirty-six hours after its use was commenced, the passive engorgement of the lungs had so much increased that the patient appeared in a hopeless condition.—At this time the attention of the class was called to the patient, and the symptoms and treatment carefully reviewed. Prof. Davis expressed the opinion that the engorged condition of the lungs was altogether passive, resulting, like the delirium, the sub-sultus, and the cardiac weakness, from the failure of those elementary properties which we call susceptibility and vital affinity, and in consequence of which the blood fails to make its wonted impression on the capillaries.

English and American pathologists very generally refer this state of things to a failure of *innervation*, and hence endeavor to remedy it by the free use of Alcholic and other diffusable stimulants. He was satisfied, however, that the failure of innervation in these cases, was not the *cause* of failure in the functions, but only a co-incident, and itself dependent on the alteration of the properties common to all the tissues, as just mentioned. A close scrutiny and rigid analysis, will enable the practitioner to arrange all the more grave cases of typhoid and typhus fevers into four classes. The first includes those cases in which the life of the patient is endangered from direct failure of the cerebral functions; the second, those in which the most alarming symptom is feebleness of the heart's action and impulse from an early tendency to softening of its muscular structure; the third, those in which the respiratory function is seriously impaired from early and progressive engorgement of the pulmonary capillaries; and the fourth, those in which life is endangered by the continued disease and ultimate disorganizations of portions of the mucous membrane of the intestines. The results of his own experience had led him to regard the use of alcoholic stimulants as positively beneficial in the first class of cases only. On the second and fourth, they produce little or no effect, while on the third, their influence is positively injurious. And as the case before us plainly belonged to that class, he directed the brandy punch to be discontinued.

He stated that the only hope of restoring such a case as the one before us, consisted in the adoption of some remedy, or combination of remedies, that would increase the susceptibility of the nervous and muscular structures, by which the heart's action would be invigorated, the tone of the pulmonary and other capillaries improved, and the progress of passive engorgements and softening of structures arrested. For producing these effects, we were familiar with no remedies more reliable than Strychnine and Oil of Turpentine. Hence, he directed the patient to continue the use of the emulsion in doses of a fluid drachm every four hours, and gave alternated with it a teaspoonful of the following mixture:

| | | |
|---|---|---|
| ℞ | Strychnine, | 1 gr. |
| | Nitric Acid, | ʒ i. |
| | Tinct. Opii., | ʒ ii. |
| | Water, | ℥ ii. |

Mixed. Beef-tea well salted, and milk porridge had been given the patient for nourishment, and the same was continued. At our next visit to the ward, two days after, we found all the symptoms of the patient much improved.

There were less delirium; less oppression of respiration; a fuller pulse, and less frequent evacuations from the bowels. The same remedies were continued, but the doses given at longer intervals. Three days subsequent we found the patient fairly convalescent, but very weak.

The evacuations from the bowels having become natural, the emulsion was discontinued, and the Strychnine Solution continued as a tonic three or four times a day. The patient slowly recovered his health. In reference to the use of Strychnine in continued fever, the doctor remarked, that in many cases between the fifth and fifteenth days, the impulse of the heart becomes week, the voluntary muscles unsteady, the cappillary circulation feeble, with an evident tendency to passive congestions in some of the internal viscera; and in such he had seldom failed to find the remedy strikingly beneficial. This was well illustrated in a case of Typhus, in a young woman directly from an emigrant ship in New York, which was admitted to the Hospital, and brought to the notice of the class a few weeks since.

